# Real-world pharmacovigilance of risperidone: Analysis based on FAERS reports from physicians and pharmacists

**DOI:** 10.1371/journal.pone.0331983

**Published:** 2025-10-17

**Authors:** Gang Hao, Zhi Jia, Yue Zan, Xiaomin Lv, Na Li, Junyin Zhou

**Affiliations:** 1 Suzhou Vocational University, Suzhou, Jiangsu, China; 2 The Affiliated Taizhou People’s Hospital of Nanjing Medical University, Taizhou, Jiangsu, China; NYU Grossman School of Medicine: New York University School of Medicine, UNITED STATES OF AMERICA

## Abstract

**Objective:**

Risperidone, a second-generation antipsychotic widely used in clinical practice, has not been systematically evaluated for its long-term real-world adverse events. This study analyzed risperidone-related AEs reported in the U.S. Food and Drug Administration Adverse Event Reporting System (FAERS) over nearly two decades, providing a reference for clinical medication.

**Methods:**

Data on risperidone was collected from FAERS covering the period from the first quarter of 2004 to the third quarter of 2024. Signal detection was performed using the reporting odds ratio, proportional reporting ratio, Bayesian confidence propagation neural network, and multi-item gamma Poisson shrinker algorithms.

**Results:**

Among the 18,089 reports collected, a total of 27 system organ classes and 452 preferred terms were identified. Nervous system disorders, Psychiatric disorders, General disorders and administration site conditions, and Injury, poisoning and procedural complications were the primary SOCs. Additionally, Reproductive system and breast disorders, Endocrine disorders, Metabolism and nutrition disorders, and Cardiac disorders showed a strong association with risperidone, warranting heightened vigilance. This study identified a substantial number of extrapyramidal-related AEs, including propulsive gait, spasmodic dysphonia, tongue thrust, respiratory dyskinesia, oromandibular dystonia, cogwheel rigidity, and pleurothotonus. Rare cardiovascular events such as Wellens’ syndrome and endocarditis fibroplastica were also uncovered through in-depth analysis. Furthermore, AEs like paroxysmal perceptual alteration, pulmonary vein occlusion, and floppy iris syndrome, although infrequent, exhibited high signal strength. This study also identified noteworthy signals not included in the risperidone label, such as rabbit syndrome (perioral tremor), jaw clicking, and portal vein cavernous transformation, which merit further investigation.

**Conclusion:**

From the perspectives of physicians and pharmacists, this research provides a unique and robust theoretical foundation for evaluating the safety profile of risperidone and offers direction for future studies on its adverse events.

## 1 Introduction

Schizophrenia is a severe mental disorder with a complex pathogenesis, characterized by marked disturbances in thinking, cognition, emotion, and behavior, which significantly impact patients’ occupational and social functioning [1]. The lifetime prevalence of schizophrenia is approximately 1% [2]. Over the past three decades (1990–2019), its global raw prevalence and incidence have risen markedly—from 14.2 to 23.6 million and from 0.94 to 1.3 million, reflecting increases of 65% and 37%, respectively [3]. Moreover, schizophrenia is associated with substantial years of potential life lost and a reduction in average life expectancy of approximately 14.5 years [4]. Beyond its profound impact on patients and their families, this disorder places a heavy economic and healthcare burden on society [5]. As a chronic illness, long-term medication is typically required, with antipsychotic drugs being the first-line treatment option.

Risperidone is an atypical antipsychotic that effectively targets positive symptoms, including hallucinations, delusions, thought disorder, and aggressiveness [6]. Several studies have suggested that risperidone may offer certain benefits in alleviating negative symptoms—such as blunted affect, emotional withdrawal, and alogia [7]—as well as improving cognitive function [8], although the extent of these effects remains a topic of ongoing research [9]. Compared to first-generation typical antipsychotics, risperidone has a lower incidence of extrapyramidal symptoms and requires a lower effective dose [4], which has led to its widespread use in clinical practice. However, in clinical use, besides the extrapyramidal symptoms associated with dosage, common adverse events (AEs) include hyperprolactinemia, which may lead to menstrual disturbances, galactorrhea, and sexual dysfunction [10]. Additionally, risperidone can affect insulin levels, causing disturbances in glucose and lipid metabolism, and may lead to severe AEs such as agranulocytosis, liver failure, and eating disorders associated with sleepwalking and insomnia [11,12]. In 2015, Canada revised the prescribing information for risperidone, strictly limiting its use in patients with vascular and mixed-type dementia [13,14]. Given the long duration since risperidone’s market introduction and its wide usage, it is necessary to analyze its adverse effects to ensure safe and rational clinical use.

The U.S. Food and Drug Administration Adverse Event Reporting System (FAERS) provides an open and free platform for collecting and analyzing post-market drug AEs, making it a valuable resource for many researchers. However, most pharmacovigilance studies based on FAERS data do not filter the reporters, leading to an overrepresentation of consumers, which may introduce a risk of result bias [15]. This study conducted a comprehensive exploration and analysis of risperidone-related real-world AEs reported by physicians and pharmacists in FAERS from 2004 to 2024, aiming to develop a clinical safety profile of risperidone.

## 2 Methods

### 2.1 Data source

The data used in this study was sourced from FAERS, which has published post-market AE reports for drugs and therapeutic biological products received by the FDA since 2004. The database is updated quarterly and contains highly structured data, including patient demographics, drug information, AE details, patient outcomes, report sources, drug administration dates, and drug indications. We collected all risperidone-related AEs reported in the FAERS database from 2004 to the third quarter of 2024. The data retrieval was based on the drug’s generic name, “Risperidone.” Given that the FAERS database is publicly accessible, and the patient records are anonymized and de-identified, this study did not require informed consent or ethical approval.

### 2.2 Data extraction and analysis

Duplicate reports were removed. In cases where multiple AE reports shared the same case ID, only the latest FDA report date (FDA_DT) was retained, and the report with the higher primary ID was selected. Only reports with a Preferred Term (PT) count greater than or equal to 3 were subjected to further analysis. The risperidone AEs selected from the FAERS database include various key pieces of information, such as patient demographics (age, gender, weight), report date, reporting country, reporter’s profession, suspected drug, type of AE, and severity. All AEs in the reports were standardized using the System Organ Classification (SOC) and PT terms from the Medical Dictionary for Regulatory Activities (MedDRA).

### 2.3 Statistical analysis

Disproportionality analysis was employed to identify and assess the signal strength of AEs related to risperidone. By combining the advantages of the Reporting Odds Ratio (ROR), Proportional Reporting Ratio (PRR), Bayesian Confidence Propagation Neural Network (BCPNN), and Multi-item Gamma Poisson Shrinker (MGPS) models, we conducted a comprehensive signal analysis. ROR and PRR are simple and intuitive methods for detecting early signals. The BCPNN method accounts for reporting uncertainty and provides the confidence level for each prediction. The MGPS algorithm, through polynomial regression and Bayesian adjustment, offers more precise detection of multidimensional and rare signals [16]. Table 1 presents the 2 × 2 contingency table, along with detailed formulas for these analysis methods and positive signal thresholds. Signal strength refers to the calculated values of ROR, PRR, IC, and EBGM. The higher the value, the stronger the signal strength, indicating a more significant association between the target drug and AE, thus reflecting a higher potential risk for the drug’s adverse reactions. All data processing was performed using Excel 2016 and MySQL 8.0, while statistical and visualization analyses were conducted using R 4.4.1.

**Table 1:** A two-by-two contingency table and detailed formulas for disproportionality analysis.

	Risperidone-related AEs	Other AEs	Sums
Risperidone	a	b	a + b
Other drugs	c	d	c + d
Sums	a + c	b + d	a + b + c + d
Algorithms	Calculation formulas		Threshold
ROR	$ROR=\frac{ad}{bc}$		a ≥ 3 and 95% CI (lower limit)>1
	$\text{9}\text{5}\%CI=e^{\text{ln}(ROR)\pm \text{1}.\text{9}\text{6}\sqrt{\frac{\text{1}}{a}+\frac{\text{1}}{b}+\frac{\text{1}}{c}+\frac{\text{1}}{d}}}$		
PRR	$PRR=\frac{a(c+d)}{c(a+b)}$		a ≥ 3 and 95% CI (lower limit)>1
	$\text{9}\text{5}\%CI=e^{\text{ln}(PRR)\pm \text{1}.\text{9}\text{6}\sqrt{\frac{\text{1}}{a}-\frac{\text{1}}{a+b}+\frac{\text{1}}{c}-\frac{\text{1}}{c+d}}}$		
BCPNN	$\chi^{\text{2}}=\frac{\left[(ad-bc{)}^{\text{2}}\right](a+b+c+d)}{\left[(a+b)(c+d)(a+c)(b+d)\right]}$		IC025 > 0
	$IC={\text{log}}_{\text{2}}\frac{a(a+b+c+d)}{\left(a+c)(a+b\right)}$		
	$E(IC)={\text{log}}_{\text{2}}\frac{\left(a+\gamma \text{1}\text{1})(a+b+c+d+\alpha )(a+b+c+d+\beta \right)}{\left(a+b+c+d+\gamma )(a+b+\alpha \text{1})(a+c+\beta \text{1}\right)}$		
	$V(IC)=\frac{\text{1}}{{\left(ln\text{2}\right)}^{\text{2}}}\{\left[\frac{(a+b+c+d)-a+\gamma -\gamma \text{1}\text{1}}{\left(a+\gamma \text{1}\text{1})(\text{1}+a+b+c+d+\gamma \right)}\right]+\left[\frac{(a+b+c+d)-(a+b)+\alpha -\alpha \text{1}}{\left(a+b+\alpha \text{1})(\text{1}+a+b+c+d+\alpha \right)}\right]+\left[\frac{(a+b+c+d)-(a+c)+\beta -\beta \text{1}}{\left(a+c+\beta \text{1})(\text{1}+a+b+c+d+\beta \right)}\right]\}$		
	$\text{9}\text{5}\%CI=IC\text{0}\text{2}\text{5}=E(IC)-\text{2}\sqrt{V(IC)}$		
	$\gamma =\gamma \text{1}\text{1}\frac{\left(a+b+c+d+\alpha )(a+b+c+d+\beta \right)}{\left(a+b+\alpha \text{1})(a+c+\beta \text{1}\right)}$		
MGPS	$EBGM=\frac{a(a+b+c+d)}{\left(a+c)(a+b\right)}$		EBGM05 > 2
	$\text{9}\text{5}\%CI=EBGM\text{0}\text{5}=e^{\text{ln}(EBGM)\pm \text{1}.\text{9}\text{6}\sqrt{\frac{\text{1}}{a}+\frac{\text{1}}{b}+\frac{\text{1}}{c}+\frac{\text{1}}{d}}}$		

### 2.4 Ethics approval and consent to participate

Ethical approval was not required for this study, as all data were obtained from the publicly accessible FAERS database and contained no personally identifiable patient information.

### 2.5 Data acquisition and participant information

The data was accessed on January 9, 2025, and the information of individual participants cannot be identified during or after data collection.

## 3 Results

### 3.1 Basic information on adverse events associated with risperidone

**Fig 1:**
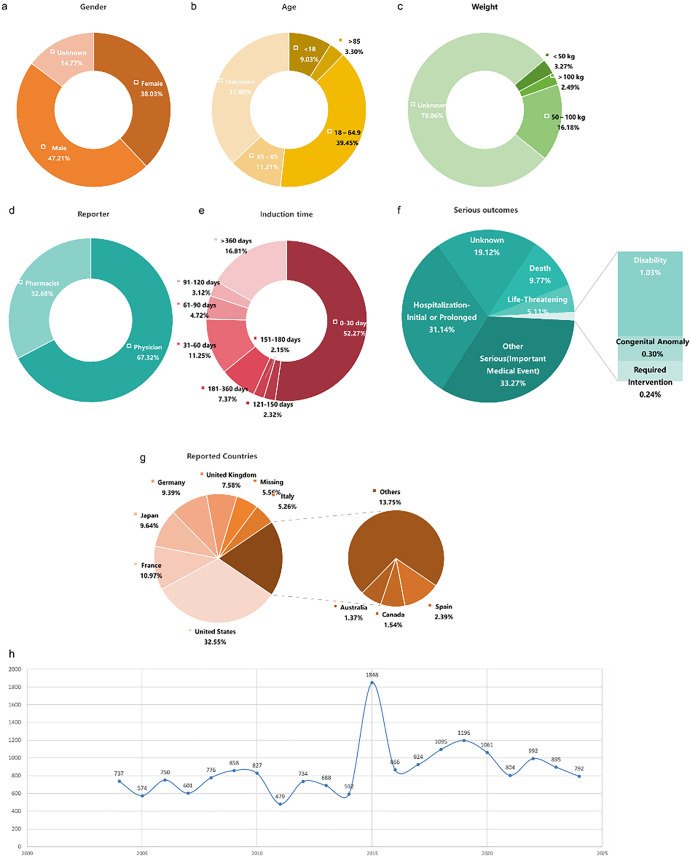
Basic Information on AE reports for Risperidone.

Between 2004 and 2024, the FAERS database recorded 18,089 AEs related to risperidone (Supplementary File S1 Table). Among these, male patients accounted for 8,539 cases (47.21%), which was significantly higher than the 6,879 cases (38.03%) reported for female patients. In terms of age distribution, 7,137 cases (39.45%) were between 18 and 65 years old, while 6,693 cases (37%) lacked age information. Furthermore, most reports (78.06%) did not include patient weight, which somewhat limits our ability to explore the impact of weight on AEs in detail. The number of reports submitted by physicians was twice as high as those submitted by pharmacists. Regarding the timing of AEs, 52.27% occurred within one month of drug use, while 16.81% appeared after one year. The Weighted Signal Proportion analysis in Table 2 indicates that the clinical signal of risperidone exhibits an early decline pattern. Notably, among severe AEs, “Other Serious, Important Medical Events” accounted for the largest proportion (33.27%), followed by hospitalization (31.14%). Deaths constituted 9.77%, and life-threatening events accounted for 5.11%. These data underscore the importance of closely monitoring potential serious AEs during risperidone treatment. Geographically, the United States had the highest number of reports, followed by France. In terms of yearly trends, the number of reports peaked in 2015, with other years remaining relatively stable (Fig 1).

**Table 2:** Time-to-onset analysis for signals with risperidone.

Prioritization				Weibull distribution				Failure type
	Case	TTO (days)		Scale parameter		Shape parameter		
	n	Median (IQR)	Min–max	α	95%CI	β	95%CI	
Risperidone	2374	26 (162)	1-13184	101.46	92.40-111.41	0.46	0.44-0.47	Early failure

Note: n, number of cases with available time-to-onset; TTO, time-to-onset; IQR, interquartile range.

### 3.2 Signal mining results

The positive signal proportions of risperidone-related AEs at the SOC level are shown in Table 3, with events distributed across 27 different SOCs. The four most significant SOCs with the highest number of reports were nervous system disorders (n = 7,842, ROR 2.13, PRR 1.95, EBGM 1.94, IC 0.96), psychiatric disorders (n = 7,563, ROR 3.65, PRR 3.24, EBGM 3.22, IC 1.69), general disorders and administration site conditions (n = 5,866, ROR 0.75, PRR 0.78, EBGM 0.78, IC −0.35), and injury, poisoning, and procedural complications (n = 5,298, ROR 1.29, PRR 1.26, EBGM 1.26, IC 0.33), which align with risperidone’s classification in the nervous system category. Additionally, we identified stronger associations with risperidone in reproductive system and breast disorders (n = 941, ROR 3.34, PRR 3.30, EBGM 3.27, IC 1.71), endocrine disorders (n = 466, ROR 2.78, PRR 2.77, EBGM 2.75, IC 1.46), metabolism and nutrition disorders (n = 1,618, ROR 1.28, PRR 1.27, EBGM 1.27, IC 0.34), and cardiac disorders (n = 2,048, ROR 1.15, PRR 1.15, EBGM 1.15, IC 0.20). Although these AEs are not characteristic of risperidone’s primary effects, their strong association with the drug highlights the need for heightened vigilance.

**Table 3:** Signal strength of AEs reports for Risperidone at the SOC level in the FAERS database.

System organ class	Case reports	ROR (95%CI)	PRR (95%CI)	χ2	EBGM (EBGM05)	IC (IC025)
Nervous system disorders	7842	2.13(2.08-2.18)	1.95(1.93, 1.97)	3931.78	1.94(1.91)	0.96(0.92)
Psychiatric disorders	7563	3.65(3.56-3.74)	3.24(3.22, 3.27)	12195.96	3.22(3.15)	1.69(1.65)
General disorders and administration site conditions	5877	0.75(0.73-0.77)	0.78(0.76, 0.81)	415.93	0.78(0.77)	−0.35(−0.39)
Injury, poisoning and procedural complications	5298	1.29(1.25-1.33)	1.26(1.23, 1.29)	308.86	1.26(1.23)	0.33(0.29)
Investigations	3716	1.07(1.04-1.11)	1.07(1.04, 1.10)	17.60	1.07(1.04)	0.10(0.05)
Cardiac disorders	2048	1.15(1.10-1.21)	1.15(1.11, 1.19)	40.28	1.15(1.11)	0.20(0.13)
Gastrointestinal disorders	1808	0.42(0.40-0.44)	0.44(0.39, 0.48)	1422.93	0.44(0.42)	−1.19(−1.26)
Product issues	1633	2.68(2.55-2.82)	2.63(2.58, 2.68)	1654.32	2.61(2.51)	1.39(1.31)
Metabolism and nutrition disorders	1618	1.28(1.21-1.34)	1.27(1.22, 1.32)	93.21	1.27(1.21)	0.34(0.27)
Respiratory, thoracic and mediastinal disorders	1440	0.54(0.51-0.57)	0.55(0.50, 0.61)	541.68	0.56(0.53)	−0.85(−0.92)
Musculoskeletal and connective tissue disorders	1132	0.46(0.44-0.49)	0.48(0.42, 0.53)	686.37	0.48(0.45)	−1.07(−1.16)
Blood and lymphatic system disorders	1081	0.75(0.71-0.80)	0.76(0.70, 0.82)	86.91	0.76(0.72)	−0.40(−0.49)
Vascular disorders	1070	0.90(0.84-0.95)	0.90(0.84, 0.96)	12.75	0.90(0.85)	−0.16(−0.24)
Infections and infestations	996	0.33(0.31-0.35)	0.34(0.28, 0.40)	1342.78	0.34(0.32)	−1.55(−1.64)
Reproductive system and breast disorders	941	3.34(3.13-3.56)	3.30(3.23, 3.36)	1496.88	3.27(3.10)	1.71(1.61)
Skin and subcutaneous tissue disorders	819	0.30(0.28-0.32)	0.31(0.24, 0.38)	1309.98	0.31(0.30)	−1.68(−1.78)
Renal and urinary disorders	798	0.80(0.75-0.86)	0.81(0.74, 0.88)	37.28	0.81(0.76)	−0.31(−0.41)
Surgical and medical procedures	696	1.17(1.09-1.26)	1.17(1.10, 1.24)	17.28	1.17(1.10)	0.23(0.12)
Eye disorders	664	0.69(0.64-0.75)	0.69(0.62, 0.77)	90.52	0.70(0.65)	−0.52(−0.64)
Hepatobiliary disorders	635	0.89(0.82-0.96)	0.89(0.81, 0.97)	8.51	0.89(0.84)	−0.17(−0.28)
Endocrine disorders	466	2.78(2.54-3.05)	2.77(2.67, 2.86)	522.13	2.75(2.55)	1.46(1.32)
Neoplasms benign, malignant and unspecified (incl cysts and polyps)	279	0.16(0.15-0.19)	0.17(0.05, 0.29)	1172.05	0.17(0.15)	−2.56(−2.73)
Social circumstances	250	1.70(1.50-1.92)	1.69(1.57, 1.82)	70.84	1.69(1.52)	0.76(0.57)
Pregnancy, puerperium and perinatal conditions	222	0.90(0.79-1.03)	0.90(0.77, 1.04)	2.25	0.90(0.81)	−0.14(−0.34)
Congenital, familial and genetic disorders	145	0.88(0.75-1.03)	0.88(0.72, 1.04)	2.43	0.88(0.77)	−0.19(−0.43)
Immune system disorders	122	0.18(0.15-0.22)	0.18(0.01, 0.36)	444.61	0.19(0.16)	−2.43(−2.69)
Ear and labyrinth disorders	79	0.44(0.35-0.54)	0.44(0.22, 0.66)	57.42	0.44(0.36)	−1.19(−1.51)

**Fig 2:**
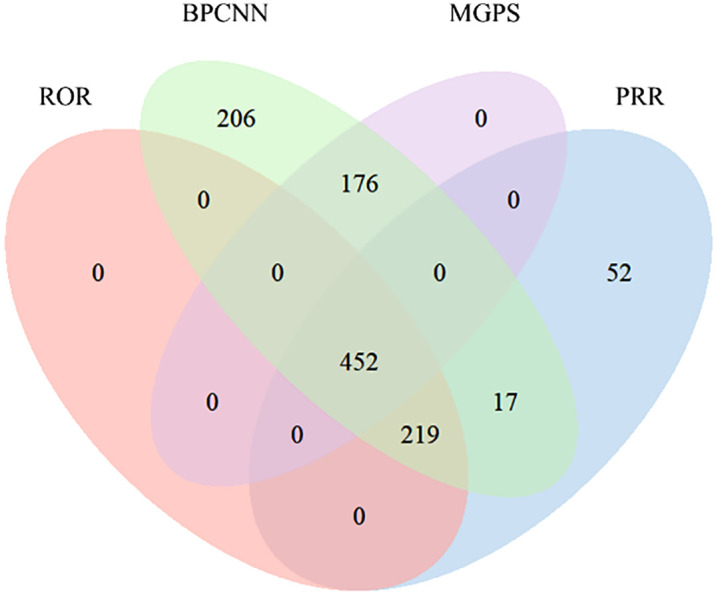
The Venn diagram of the four different algorithms (Among the 3054 signal combinations, the ROR method identified 671 related signals, the PRR method identified 740, the MGPS method identified 628, and the BCPNN method identified 1070 valid signals.).

At the PT level, a total of 452 risperidone-related AE signals were detected based on the four algorithms (Fig 2). Table 4 presents the top 30 PTs ranked according to the EBGM algorithm, with a relatively even distribution across SOCs. In this study, the top three highest signal strength AEs were device use confusion (n = 41, ROR 311.93, PRR 311.67, EBGM 152.45, IC 7.25), exposure to contaminated device (n = 22, ROR 250.97, PRR 250.86, EBGM 136.34, IC 7.09), and Wellens’ syndrome (n = 8, ROR 237.21, PRR 237.17, EBGM 132.21, IC 7.05). The three AEs with the highest number of reports were blood prolactin increased (n = 307, ROR 72.80, PRR 72.35, EBGM 58.35, IC 5.87), hyperprolactinaemia (n = 287, ROR 83.90, PRR 83.42, EBGM 65.32, IC 6.03), and galactorrhoea (n = 205, ROR 56.19, PRR 55.96, EBGM 47.24, IC 5.56). After excluding AEs related to disease progression and PTs unrelated to the drug, the study also identified rare adverse reactions such as endocarditis fibroplastica. Moreover, although the occurrence of paroxysmal perceptual alteration (PPA), pulmonary vein occlusion, and floppy iris syndrome was relatively low, their signal strength was notably high. Importantly, we discovered several adverse reactions not included in the drug’s official labeling, such as rabbit syndrome, jaw clicking, and portal vein cavernous transformation, some of which exhibit high signal strength and warrant clinical attention.

**Table 4:** The top 30 signal strength of AEs reports for Risperidone at the PT level.

SOC	PTs	Case reports	ROR (95% CI)	PRR (95% CI)	χ2	EBGM (EBGM05)	IC (IC025)
Injury, poisoning and procedural complications	Device use confusion	41	311.93 (201.2-483.61)	311.67 (311.23-312.11)	6189.61	152.45 (105.63)	7.25 (6.7)
Injury, poisoning and procedural complications	Exposure to contaminated device	22	250.97 (142.23-442.82)	250.86 (250.29-251.42)	2965.59	136.34 (84.78)	7.09 (6.36)
Cardiac disorders	Wellens’ syndrome	8	237.21 (93.62-601.06)	237.17 (236.24-238.1)	1045.23	132.21 (60.73)	7.05 (5.88)
General disorders and administration site conditions	Propulsive gait	3	177.89 (42.51-744.4)	177.88 (176.45-179.31)	329.78	111.55 (33.68)	6.80 (5.07)
Surgical and medical procedures	Therapeutic hypothermia	4	169.42 (49.59-578.78)	169.41 (168.18-170.64)	426.15	108.17 (38.7)	6.76 (5.22)
Pregnancy, puerperium and perinatal conditions	Somatic symptom disorder of pregnancy	6	161.73 (59.81-437.33)	161.71 (160.71-162.7)	620.07	104.99 (45.67)	6.71 (5.43)
Psychiatric disorders	Paroxysmal perceptual alteration	5	148.25 (50.67-433.75)	148.23 (147.16-149.31)	487.47	99.16 (40.38)	6.63 (5.25)
Nervous system disorders	Rabbit syndrome	24	134.31 (82.92-217.56)	134.25 (133.77-134.73)	2184.81	92.72 (61.93)	6.53 (5.87)
Social circumstances	Homosexuality	4	118.6 (37.19-378.16)	118.59 (117.43-119.75)	333.13	84.99 (32.21)	6.41 (4.92)
Injury, poisoning and procedural complications	Dermatitis artefacta	3	98.83 (26.75-365.07)	98.82 (97.52-100.13)	217.87	74.37 (24.92)	6.22 (4.57)
Musculoskeletal and connective tissue disorders	Jaw clicking	6	93.63 (37.39-234.46)	93.62 (92.7-94.54)	417.84	71.39 (33.12)	6.16 (4.93)
Nervous system disorders	Spasmodic dysphonia	19	89.44 (53.55-149.41)	89.41 (88.90-89.92)	1276.15	68.93 (44.87)	6.11 (5.39)
Cardiac disorders	Endocarditis fibroplastica	5	87.20 (32.17-236.38)	87.20 (86.02-88.19)	329.21	67.61 (29.35)	6.08 (4.76)
Endocrine disorders	Hyperprolactinaemia	287	83.90 (73.58-95.67)	83.42 (83.29-83.55)	18240.06	65.32 (58.53)	6.03 (5.84)
Nervous system disorders	Pleurothotonus	78	75.69 (59.02-97.07)	75.57 (75.32-75.82)	4573.76	60.42 (49.07)	5.92 (5.56)
Investigations	Blood prolactin increased	307	72.80 (64.24-82.5)	72.35 (72.22-72.47)	17365.78	58.35 (52.55)	5.87 (5.69)
Hepatobiliary disorders	Portal vein cavernous transformation	3	68.42 (19.50-240.11)	68.42 (67.16-69.67)	161.92	55.77 (19.51)	5.80 (4.20)
Injury, poisoning and procedural complications	Skin pressure mark	5	67.39 (25.52-177.95)	67.38 (66.41-68.35)	266.42	55.09 (24.44)	5.78 (4.49)
Infections and infestations	Bed bug infestation	5	64.46 (24.5-169.55)	64.45 (63.48-65.42)	256.55	53.12 (23.65)	5.73 (4.44)
Gastrointestinal disorders	Tongue thrust	3	63.53 (18.26-221.09)	63.53 (62.28-64.78)	152.05	52.49 (18.49)	5.71 (4.12)
Nervous system disorders	Respiratory dyskinesia	4	62.42 (21.23-183.49)	62.41 (61.34-63.49)	199.68	51.73 (20.99)	5.69 (4.27)
Psychiatric disorders	Waxy flexibility	9	58.01 (28.4-118.53)	58.00 (57.29-58.72)	421.69	48.68 (26.77)	5.61 (4.61)
Reproductive system and breast disorders	Galactorrhoea	205	56.19 (48.39-65.25)	55.96 (55.81-56.11)	9309.65	47.24 (41.68)	5.56 (5.34)
Metabolism and nutrition disorders	Diabetes with hyperosmolarity	3	55.59 (16.2-190.79)	55.59 (54.35-56.82)	135.42	46.97 (16.74)	5.55 (3.97)
Respiratory, thoracic and mediastinal disorders	Pulmonary vein occlusion	6	53.91 (22.59-128.67)	53.90 (53.03-54.77)	263.60	45.76 (22.1)	5.52 (4.33)
Nervous system disorders	Oromandibular dystonia	33	50.99 (35.24-73.78)	50.96 (50.59-51.32)	1379.15	43.63 (32.03)	5.45 (4.92)
Nervous system disorders	Cogwheel rigidity	52	44.35 (33.13-59.36)	44.30 (44.01-44.59)	1914.70	38.67 (30.3)	5.27 (4.85)
Injury, poisoning and procedural complications	Floppy iris syndrome	14	42.36 (24.2-74.17)	42.35 (41.79-42.91)	494.61	37.18 (23.27)	5.22 (4.42)
Metabolism and nutrition disorders	Ketosis-prone diabetes mellitus	3	42.35 (12.63-142)	42.35 (41.14-43.56)	105.99	37.18 (13.51)	5.22 (3.66)
Skin and subcutaneous tissue disorders	Target skin lesion	6	41.37 (17.61-97.21)	41.37 (40.51-42.22)	207.41	36.42 (17.82)	5.19 (4.02)

## 4 Discussion

Risperidone is a benzisoxazole derivative and, as a second-generation antipsychotic, has a high affinity for the 5-HT_2A_ receptor and dopamine D_2_ receptor. In addition, risperidone exhibits significant affinity for dopamine D_3_ and D_4_ receptors, α-adrenergic receptors, and H_1_ histamine receptors, but does not bind to cholinergic receptors [17]. Its unique receptor profile contributes to its widespread clinical application. However, with increasing use, multiple studies and clinical practices have shown that risperidone can lead to hyperprolactinemia [18], sexual dysfunction [10], endocrine effects [19], and granulocytopenia [20]. Therefore, the safety profile of risperidone is of significant concern. Routine FAERS analyses are at risk of result bias to some extent, as a considerable proportion of AE reports are submitted by non-medical professionals. This study, from the perspective of pharmacists and physicians, provides a comprehensive analysis of the FAERS database over the past two decades to reveal AEs associated with risperidone, aiming to depict its safety profile for clinical reference.

This study shows that the number of AE reports is higher in males than in females, which may be attributed to the higher incidence of schizophrenia in males (M/F = 1.1), leading to higher usage rates and reporting rates [3]. It may also be related to the lower drop-out rate among male patients using risperidone [21]. We found that the highest proportion of AE reports came from the 18–65 age group, followed by the 65–85 age group. This may be related to the higher prevalence in this population. Schizophrenia tends to occur more frequently in early adulthood, peaking around the age of 40. Males reach the peak incidence in their 20s, with a gradual decline afterward, while females show a gradual increase in incidence after 40, surpassing males and reaching a second peak in later years [22]. The reported age distribution may reflect the two peaks of treatment rather than an inherently increased risk of AEs compared to other age groups. Although patients weighing 50–100 kg accounted for the largest proportion, 78.06% of reports did not specify the patients’ weight, limiting our ability to interpret the differences in AE occurrence based on weight groups. Notably, physicians accounted for two-thirds of the reports, highlighting the active role of doctors in AE reporting. In terms of AE onset, more than half of the events occurred within one month of drug use, but 16.81% were reported after one year, suggesting that clinicians should conduct long-term follow-up and assessments when using risperidone, as AEs may not manifest until after some time. Regarding severe outcomes, a relatively high proportion of reports involved hospitalizations or extended hospital stays, with “Other Serious (Important Medical Event)” being reported frequently, requiring close attention. Time series analysis showed that the number of AE reports peaked in 2015 and gradually declined thereafter, which may be related to the long-term clinical use of the drug and the growing familiarity of the public and healthcare professionals with the drug’s safety profile. In terms of report sources, the highest proportion of AE reports came from the United States, likely due to FAERS being a U.S.-based AE reporting database, but this also suggests that the data may be biased and not fully represent differences across various ethnic groups.

Our study found that the AEs related to risperidone were primarily concentrated in systems such as nervous system disorders, psychiatric disorders, and general disorders and administration site conditions, which are consistent with the pharmacological mechanism of risperidone. Additionally, AEs in systems like reproductive system and breast disorders, endocrine disorders, metabolism and nutrition disorders, and cardiac disorders showed higher signal strength, consistent with current clinical practice. Risperidone affects multiple neuroendocrine axes, with hyperprolactinemia being one of the more common manifestations [10]. Clinical manifestations include amenorrhea, galactorrhea, sexual dysfunction, and infertility [18]. The primary mechanism involves potent antagonism of D₂ receptors in the tuberoinfundibular pathway, which removes dopaminergic inhibition on pituitary lactotrophs and promotes prolactin secretion [23]. Preclinical studies further indicate that risperidone downregulates dopamine synthesis in the hypothalamic arcuate nucleus by modulating neuropeptide Y and tyrosine hydroxylase gene expression, thereby amplifying the disinhibition of prolactin release [24]. Positron emission tomography (PET) studies confirm a strong correlation between pituitary D_2_ receptor occupancy and serum prolactin levels, supporting D_2_ blockade as the central mechanism of this adverse effect [25]. Chronic hyperprolactinemia suppresses luteinizing hormone and follicle-stimulating hormone secretion, leading to reduced estrogen or testosterone levels [26], which increases the risk of osteoporosis and fractures [27].

Moreover, risperidone may affect the hypothalamic–pituitary–thyroid (HPT) axis. Studies have reported that risperidone users may show elevated thyroid-stimulating hormone (TSH) levels along with reduced thyroxine and triiodothyronine [28]. These findings suggest that risperidone might interfere with the HPT axis [29] or enhance TSH secretion through dopamine antagonism, thereby affecting thyroid hormone synthesis and peripheral metabolism [30]. However, the precise mechanism remains unclear.

Regarding metabolism, current evidence is mixed. Most studies indicate that risperidone significantly affects glucose–lipid metabolism and energy balance. Its antagonism of 5-HT_2C_ [31] and H_1_ receptors [32] increases appetite and promotes fat accumulation, while the melanocortin-4 receptor pathway may also contribute to risperidone-induced metabolic syndrome [33]. Long-term use has been associated with insulin resistance (elevated HOMA-IR), increased fasting glucose, higher triglycerides, body fat percentage, and body mass index, which collectively raise the risk of metabolic syndrome and cardiovascular events [34]. However, one long-term study in male rats found reduced food intake and body weight after chronic risperidone administration [19]. Nevertheless, the existing literature consistently emphasizes its endocrine effects, although the exact mechanism remains to be further elucidated. Additionally, several studies suggest that risperidone may influence the hypothalamic–pituitary–adrenal (HPA) axis by modulating cortisol levels and cytokines such as IL-2 and IL-6 [35].

At the PT level, although extrapyramidal symptoms (EPS) were not directly mentioned, our study identified a considerable proportion of various EPS, such as propulsive gait, spasmodic dysphonia, tongue thrust, respiratory dyskinesia, oromandibular dystonia, and cogwheel rigidity, including some rare EPS like pleurothotonus, all of which exhibited strong signal strength. Research indicates that, although risperidone has a relatively weak anticholinergic adverse effect, it is considered one of the second-generation antipsychotic drugs with the highest risk of inducing extrapyramidal symptoms [36]. Additionally, symptoms like cogwheel rigidity—typically associated with Parkinson’s disease psychosis or parkinsonism [37] —may be confused with the spasticity seen in serotonin syndrome [38], complicating clinical differentiation. These findings all point to a same conclusion: although risperidone provides unprecedented clinical benefits for schizophrenia, its EPS risk requires close attention. This finding may extend previous insights into risperidone, particularly for patients with comorbid Parkinson’s disease.

Wellens’ syndrome and endocarditis fibroplastica are rare cardiovascular AEs that deserve particular attention. Wellens’ syndrome, also known as anterior descending T-wave syndrome, is characterized by deep, symmetrical, inverted T-waves in the chest leads of the electrocardiogram (ECG) without significant ST-segment deviation [39]. This may be related to risperidone’s blockage of potassium ion channels, which causes electrophysiological abnormalities in myocardial cells, leading to prolonged myocardial repolarization and resulting in ECG changes such as T-wave inversion or bidirectional alterations. Additionally, risperidone’s high affinity for the 5-HT_2_ receptor may contribute to cardiac conduction abnormalities, potentially triggering Wellens’ syndrome [40]. It is important to note that, in addition to Wellens’ syndrome, risperidone carries an increased risk of arrhythmias. This may be due to risperidone’s inhibition of α1 and α2 receptors, which enhances myocardial contractility and causes excessive cardiac excitation. It can also increase the risk of arrhythmias by inhibiting Na + -K + -ATPase in myocardial cells, damaging cell integrity and altering membrane permeability [41,42]. Endocarditis fibroplastica is a rare disease characterized by eosinophil proliferation and infiltration of myocardial cells, leading to myocardial cell necrosis and endocardial fibrosis. The exact mechanism is unclear, but a potential explanation is that antipsychotic drugs like risperidone may indirectly promote eosinophil activation by affecting immune systems or metabolic pathways, thereby increasing the risk of endocarditis fibroplastica [43]. These findings reveal previously unrecognized risks of risperidone, warranting further investigation.

Although the occurrence is infrequent, PPA exhibit high signal strength. PPA refers to brief, recurrent episodes of altered perception, first described by Yamaguchi in 1985 [44], with an estimated prevalence of 3.25%−4.0% [45,46]. It is characterized by perceptual hypersensitivity—particularly heightened sensitivity to visual stimuli—along with psychedelic-like experiences and disturbances in body schema [46]. Although the exact pathophysiology remains unclear, dopaminergic imbalance induced by antipsychotics is considered a key factor [47]. Specifically, inhibition of the mesolimbic and mesocortical dopaminergic systems, along with compensatory activation of the noradrenergic system, is thought to be associated with PPA [46]. Within the visual system, dopamine deficiency in the retina and visual cortex may impair visual processing, including contrast sensitivity [48]. While rare and limited in literature, PPA has been observed not only with risperidone [47,49] but also with other psychotropic agents, including haloperidol [48], clozapine [50], and lithium carbonate [51]. The relatively higher incidence of PPA with risperidone may be attributed to its strong and sustained binding affinity for D_2_ receptors [46]. Moreover, PPA has also been reported in association with oculogyric crisis [52].

Pulmonary vein occlusion and floppy iris syndrome also demonstrated high signal strength. Risperidone may affect blood coagulation and blood flow, increasing the risk of thrombus formation and leading to pulmonary vein occlusion [53]. It is also considered to carry a risk of pulmonary embolism [54]. Additionally, it can cause eye muscle control dysfunction, resulting in floppy iris syndrome [55]. Furthermore, the strongest signals were observed for “device use confusion” and “exposure to contaminated device”, which may be associated with errors in handling or administering the long-acting injectable microsphere formulation of risperidone. Such issues could include incorrect reconstitution, improper injection technique, or storage errors. Clinicians should be aware of these potential risks when prescribing risperidone, perform appropriate assessments before and after administration, and implement preventive measures to identify and manage such complications early.

Importantly, we have identified adverse reactions that are not mentioned in the product label, such as rabbit syndrome, jaw clicking, and portal vein cavernous transformation. Rabbit syndrome, now referred to as perioral tremor, is a tardive extrapyramidal side effect caused by long-term use of medications, especially antipsychotics [56]. It was first reported by Villeneuve in 1972 [57]. The pathophysiological mechanism of rabbit syndrome remains unclear, but it is believed to result from the antipsychotic-induced blockade of extrapyramidal dopaminergic neurons, leading to a hypercholinergic state [58] and ultimately disrupting normal basal ganglia function [56]. The similarity and potential confusion between rabbit syndrome and tardive dyskinesia warrant close attention [59]. Jaw clicking is a bothersome symptom, with approximately 70% of initially painless patients eventually developing pain [60,61]. Although the mechanism is not well understood, changes in adrenaline levels caused by risperidone may be related to this symptom [62]. Portal vein cavernous transformation is a condition caused by partial or complete obstruction of the portal vein, commonly seen in patients with cirrhosis or portal vein thrombosis. Despite the strong signal, further research is needed to confirm the causal relationship between risperidone and portal vein cavernous transformation. These risk signals may indicate unknown neural pathways associated with the drug, requiring further clinical attention and research.

## 5 Limitation

This study, from the perspective of doctors and pharmacists, provides a comprehensive analysis of the real-world AEs associated with risperidone over the past two decades. It identifies multiple signals that warrant attention and explores potential mechanisms, offering valuable references for the clinical application of the drug. However, certain limitations are inevitably present. First, as a spontaneous reporting system, FAERS is subject to underreporting and misreporting. Mild AEs are less likely to be reported, whereas serious events are more likely to be reported, introducing potential reporting bias. For example, the bed bug infestation found in the results should be interpreted carefully. Second, FAERS does not provide the total number of patients exposed to specific drugs, making it impossible to calculate the true incidence. Third, FAERS cannot establish causal relationships between drugs and AEs. Disproportionality analyses require comprehensive and accurate data, and their interpretation may be challenging when multiple variables or confounders are involved. For instance, patients treated with risperidone may systematically differ from those receiving other antipsychotics in terms of comorbidities or concomitant medications. Consequently, some reported events may be attributable to these underlying patient characteristics.

Moreover, the analysis did not stratify by risperidone dose, route of administration, or treatment duration, which may affect the interpretation of dose- or time-dependent AE patterns. Additionally, due to the nature of spontaneous reporting in FAERS, the diagnostic basis for certain rare and complex AEs (e.g., Wellens’ syndrome, rabbit syndrome) is often unclear, which may lead to potential misclassification and should be interpreted with caution. Finally, in our study, the majority of reports originated from the United States and France. Therefore, the generalizability of the findings to non-Western populations may be limited due to potential differences in genetic background, dietary habits, and healthcare systems. Future research should combine multi-center prospective studies with epidemiological research to further explore the safety profile of risperidone.

## 6 Conclusion

As a long-term pharmacovigilance analysis of risperidone based on the FAERS database, we have identified several common and rare AEs associated with risperidone from the perspectives of doctors and pharmacists, providing a solid scientific foundation for the safety evaluation of risperidone. A significant proportion of extrapyramidal symptoms in our study raised notable concern, and some rare cardiovascular AEs, such as Wellens’ syndrome and endocarditis fibroplastica, warrant further investigation. Additionally, while the occurrence of paroxysmal perceptual alteration, pulmonary vein occlusion, and floppy iris syndrome is low, their high signal strength still requires attention. New adverse reactions such as rabbit syndrome, jaw clicking, and portal vein cavernous transformation should not be overlooked, and the mechanisms behind them deserve further exploration. These AEs collectively highlight the complex impact of risperidone on patient health, suggesting that despite its long market presence, close monitoring of risperidone use in clinical practice is essential to ensure patient safety.

## Supporting information

S1 Table Basic Information on AE reports for Risperidone. (DOCX)

S1 File Risperidone.zip. Raw data of risperidone.
(ZIP)
