# Supplementary material for: Real-world pharmacovigilance of risperidone: Analysis based on FAERS reports from physicians and pharmacists
Source: PLoS One. 2025 Oct 17;20(10):e0331983. doi: 10.1371/journal.pone.0331983 (PMC12533923; doi:10.1371/journal.pone.0331983)
Supplement: S1 Table — (DOCX) [file pone.0331983.s001.docx]

Supplementary File

Table S1 Basic Information on AE reports for Risperidone

| Factor | Number of events (%) |
| --- | --- |
| Gender |  |
| Female | 6879 (38.0%) |
| Male | 8539 (47.2%) |
| Unknown | 2671 (14.8%) |
| Age |  |
| <18 | 1634 (9.0%) |
| 18-64 | 7137 (39.5%) |
| 65-85 | 2028 (11.2%) |
| >85 | 597 (3.3%) |
| Unknown | 6693 (37.0%) |
| Weight |  |
| ＜50 kg | 591 (3.3%) |
| 50-100kg | 2927 (16.2%) |
| >100kg | 451 (2.5%) |
| Unknown | 14120 (78.1%) |
| Reported Countries (top 10) |  |
| United States | 5888 (32.55%) |
| France | 1985 (10.97%) |
| Japan | 1744 (9.64%) |
| Germany | 1698 (9.39%) |
| United Kingdom | 1372 (7.58%) |
| Entity 1 | 1005 (5.56%) |
| Italy | 951 (5.26%) |
| Spain | 432 (2.39%) |
| Canada | 279 (1.54%) |
| Australia | 248 (1.37%) |
| Reporter |  |
| Physician | 12178 (67.3%) |
| Pharmacist | 5911 (32.7%) |
| Serious outcomes |  |
| Hospitalization-Initial or Prolonged | 5633 (31.1%) |
| Death | 1768 (9.8%) |
| Life-Threatening | 925 (5.1%) |
| Disability | 187 (1.0%) |
| Required Intervention to Prevent Permanent  Impairment/Damage | 44 (0.2%) |
| Congenital Anomaly | 54 (0.3%) |
| Unknown | 3459 (19.1%) |
| Induction time |  |
| 0-30 days | 1241 (52.27%) |
| 31-60 days | 267 (11.25%) |
| 61-90 days | 112 (4.72%) |
| 91-120 days | 74 (3.12%) |
| 121-150 days | 55 (2.32%) |
| 151-180 days | 51 (151-180 days) |
| 181-360 days | 175 (7.37%) |
| >360 days | 399 (16.81%) |
